# The Addition of a 3D Balanced Steady‐State Free Precession Pulse Sequence Improves Magnetic Resonance Imaging Identification of Certain Canine Cranial Nerves

**DOI:** 10.1111/vru.70204

**Published:** 2026-06-19

**Authors:** Rachel Durrwachter, Wilfried Mai, Alessia Cordella, Jennifer Reetz, Matthew Paek

**Affiliations:** ^1^ Department of Clinical Sciences and Advanced Medicine School of Veterinary Medicine University of Pennsylvania Philadelphia Pennsylvania USA; ^2^ VetRad Gaithersburg Maryland USA

**Keywords:** facial nerve, Gradient Echo, magnetic resonance imaging (MRI), vestibulocochlear nerve

## Abstract

In people, magnetic resonance imaging (MRI) with high‐resolution, high T2‐weighted (T2w) contrast‐balanced steady‐state free precession (b‐SSFP) pulse sequences and 3D fast spoiled gradient‐echo (FSPGR) pulse sequences can improve visualization of cranial nerves (CNs) and associated osseous foramina. This prospective, observational study aimed to determine whether the addition of b‐SSFP and FSPGR sequences improves confidence in the identification of canine CNs compared to the standard imaging protocol at 1.5 T. The head of 10 canine cadavers was imaged, including transverse T1‐weighted (T1w) and T2w fast spin echo (FSE); sagittal T2w FSE; 3D Fast Imaging Employing Steady‐State Acquisition with Cycling (FIESTA‐C); and 3D Liver Acquisition with Volume Acceleration (LAVA). Board‐certified radiologists rated their confidence in identifying CNs II–XII using a four‐point scale. Observers evaluated standard sequences alone, then with the addition of transverse FIESTA‐C, and finally with the inclusion of FIESTA‐C and transverse LAVA. Scores between protocols were compared with the Wilcoxon matched‐pairs signed‐rank test. Interobserver agreement was measured with weighted kappa. Median score increased when adding FIESTA‐C for all observers for nerves VII, VIII, IX, and X, and for two observers for nerves III and XI. Matched‐pairs comparison revealed significant differences for nerves VII and VIII for all observers, and for nerves III, IX, X, XI, and XII for two observers. Further addition of LAVA generally did not improve confidence. Interobserver agreement was overall moderate to substantial, but slight to fair for some nerves and pulse sequences. Addition of a 3D b‐SSFP sequence may improve identification of some CNs, especially VII and VIII, which are somewhat commonly affected by middle ear pathology in dogs.

## 1 Introduction

In dogs with cranial nerve (CN) deficits, magnetic resonance imaging (MRI) is often employed to confirm and further characterize CN disease. Numerous studies have documented computed tomography (CT) and MRI evaluations of CNs in veterinary species, including their normal appearance, anatomic pathways on MRI, and osseous and foraminal landmarks on CT scans [1, 2, 3, 4]. The visualization of the cisternal course of the CNs depends on, among other factors, the anatomic properties of each individual nerve, such as diameter, course, and the amount of surrounding cerebrospinal fluid (CSF). The small diameter of some of these nerves is, in many instances, the main reason for the difficulty in visualizing them with conventional MRI pulse sequences [5]. CNs II, III, V, and VIII are usually identified, whereas CNs IV, VII, IX, X, and XI are inconsistently seen, and CNs I (due to its non‐discrete nature), VI, and XII are often difficult to identify at all [1, 3, 6].

In human medicine, balanced steady‐state free precession (b‐SSFP) pulse sequences like CISS (Constructive Interference in Steady State, Siemens) and FIESTA‐C (Fast Imaging Employing Steady‐State Acquisition with Cycling, GE Healthcare) enhance visualization of the cisternal portion of the CNs by providing three‐dimensional imaging with high spatial resolution and T2‐weighted (T2w) contrast [5, 7]. This aids in identifying small hypointense structures surrounded by high signal CSF, such as the CNs [5, 7, 8, 9].

In b‐SSFP pulse sequences, the time of repetition (TR) is kept shorter than the T2 relaxation time of the tissue of interest. Therefore, there is insufficient time for the transverse magnetization to fully decay before the subsequent radiofrequency (RF) pulse, resulting in residual transverse magnetization. After several repeated TR periods, a “steady state” is established in which there is a continuous exchange between longitudinal and transverse magnetization. This produces a strong signal and excellent contrast resolution, particularly for tissues with a high T2/T1 ratio, such as CSF and fat. Additionally, these sequences are often acquired at sub‐millimetric slice thicknesses, thereby improving spatial resolution [6].

On CT studies, osseous and foraminal landmarks aid in anatomically depicting small structures, such as CNs [3]; however, these may be difficult to identify with standard MRI pulse sequences. 3D fast spoiled gradient‐echo (FSPGR) pulse sequences provide high‐resolution images with good contrast between the very low signal of bone and adjacent muscles/fatty bone marrow. Examples include Volumetric Interpolated Breath‐hold Examination (VIBE, Siemens) or Liver Acquisition with Volume Acceleration (LAVA, GE Healthcare). Ultrashort TE pulse sequences (e.g., PETRA [Pointwise Encoding Time Reduction with Radial Acquisition] on Siemens scanners) were also shown to provide images of canine skull structures equivalent to those obtained with CT [10]. These high‐resolution sequences enhance the definition of skull structures, aiding in the identification of CN pathways [6].

Although b‐SSFP pulse sequences have been shown to be beneficial for the diagnosis of selective spinal conditions in dogs (e.g., arachnoid diverticula) [11], there is no published literature regarding the use of b‐SSFP and FSPGR pulse sequences for evaluation of the canine brain and CNs. On the basis of human literature, we hypothesized that adding a 3D b‐SSFP sequence would improve confidence in the identification of multiple CNs (without specifying nerve‐specific effects, given the exploratory nature of this study in dogs). Therefore, the aims of the study were to determine whether the addition of a 3D b‐SSFP pulse sequence improved radiologists’ confidence in identifying canine CNs as compared to standard pulse sequences used for canine brain imaging. A secondary aim was to assess whether adding a sequence that improves osseous detail, such as 3D LAVA, further increases confidence in nerve visualization.

## 2 Materials and Methods

This was a prospective, observational, exploratory study design. Ten cadaveric canine specimens were used. Dogs included died or were euthanized <24 h prior to MRI examination for reasons unrelated to their neurologic status. Their cadavers were donated by the owners to an institutional educational program. Therefore, Institutional Animal Care and Use Committee approval was not necessary.

Dogs were positioned in sternal recumbency. In most dogs (*n* = 8), a 32‐channel flex coil (GEM Flex Suite, GE Healthcare) was used; in two dogs with smaller heads, where the 32‐channel coil would be too large, a 24‐channel coil was used to fit snugly and ensure an adequate signal‐to‐noise ratio. MRI of the brain was obtained in a 1.5 T scanner (Signa Explorer, GE Healthcare). Transverse T1‐weighted (T1w) and T2w fast spin echo (FSE); sagittal T2w FSE; 3D b‐SSFP (FIESTA‐C) and 3D T1w FSPGR (LAVA) sequences were acquired. A zero‐filled interpolation factor of 2 was used for 3D FIESTA‐C and 3D LAVA to increase the number of locations in the slice‐encoding direction (see Table 1 for other imaging parameters). The resulting voxel size for the 3D sequences was 0.78 × 0.78 × 0.8 mm^3^, that is, nearly isotropic.

**TABLE 1:** Magnetic resonance imaging (MRI) sequences and imaging parameters for evaluation of the brain.

Parameter	T2w sagittal	T2w transverse	T1w transverse	3D FIESTA‐C	3D‐LAVA
TR (ms)	2756–4780	2500–2699	584–655	6.01–6.52	6.55–6.98
TE (ms)	73.1–76.7	74.1–78	10.4–10.8	2.45–2.71	3.11–3.23
Number of averages	2	2	2	3	1.9
Slice thickness (mm)	2.45 (range 2–3)	2.95 (range 2.5–3)	2.95 (range 2.5–3)	0.8	0.8
Slice gap (mm)	0.3	0.5	0.5	0	0
Echo train length	14	14	3	1	1
Acquisition matrix	256 × 224	256 × 256	256 × 256	180 × 180	180 × 180
Flip angle (°)	160	160	160	60	10
Field of view (mm)	160 × 160	140 × 140	140 × 140	140 × 140	140 × 140

Abbreviations: FIESTA‐C, Fast Imaging Employing Steady‐State Acquisition with Cycling; LAVA, Liver Acquisition with Volume Acceleration; T1w, T1‐weighted; T2w, T2‐weighted; TR, time of repetition; TE, time of echo.

To ensure inclusion of relatively normal brain anatomy, patients were excluded based on any prior or current history of neurologic clinical signs. Prior to inclusion, the images were inspected by an ACVR‐board‐certified radiologist (W.M. signal) to make sure the images were adequate and free of pathology that would distort the CN pathways.

Three board‐certified radiologists (two ACVR board‐certified [M.P., J.R.] and one ECVDI board‐certified [A.C.]) were asked to rate their confidence in identifying CNs II–XII using a four‐point scale, based on the extent of nerve visualization. CN I was excluded due to its non‐discrete nature. Each pair of nerves was assessed together and assigned a grade from 0 to 3 based on the following criteria: 0 (not visualized at all); 1 (incompletely [>0% but <50%] visualized); 2 (partially [>50% but less than 100%] visualized); and 3 (completely visualized, the entire nerve can be traced from origin to exit). For CN V, observers were instructed to consider the visualization of all three branches when assigning a single score; however, no specific guidance was provided on how to weight discrepancies in branch visualization.

Observers initially graded nerves using the standard pulse sequences (T1w and T2w FSE). One month later, to minimize recall bias, they repeated grading with the transverse standard sequences and addition of the transverse b‐SSFP (3D FIESTA‐C) image series. Finally, 1 month later, the same cases were re‐evaluated and graded again, with standard transverse sequences and both the transverse 3D FIESTA‐C and the transverse 3D LAVA image series included. Although the 3D sequences have the potential to be reformatted in multiple planes, the transverse plane was chosen as it was shown in previous studies to be the best plane to identify the CNs, likely due to the fact that most nerves have a transverse or caudal‐to‐rostral trajectory after their emergence, so that they tend to be more easily identified and traced when scrolling through transverse images from caudal to rostral [3].

Statistical analyses were performed by one of the authors (W.M.) using commercially available software (StataNow/BE 19.5). Overall percent agreement between observers was computed for each CN and each pulse sequence combination. Interobserver agreement was measured with quadratic weighted Kappa, with Kappa values interpreted as follows [12]: 0.00–0.20 = slight agreement, 0.21–0.40 = fair agreement, 0.41–0.60 = moderate agreement, 0.61–0.80 = substantial agreement, and 0.81–1.00 = almost perfect agreement. For each nerve and observer, scores between pulse sequence combinations were compared with the Wilcoxon matched‐pairs signed‐rank test (standard vs. standard + FIESTA‐C; standard + FIESTA‐C vs. standard + FIESTA‐C + LAVA). *p* values <0.05 were considered significant.

## 3 Results

The mean age of the 10 dogs was 9.9 years (2–15 years old), and the mean weight was 23.9 kg (11.4–38 kg). Nine different breeds were represented, including Beagle, Pit Bull Terrier, Golden Retriever, Great Pyrenees, Husky, Australian Shepherd, German Shepherd, Dutch Shepherd, and two mixed‐breed dogs. Five dogs were female (one intact, four spayed), and five dogs were male and castrated.

**FIGURE 1:**
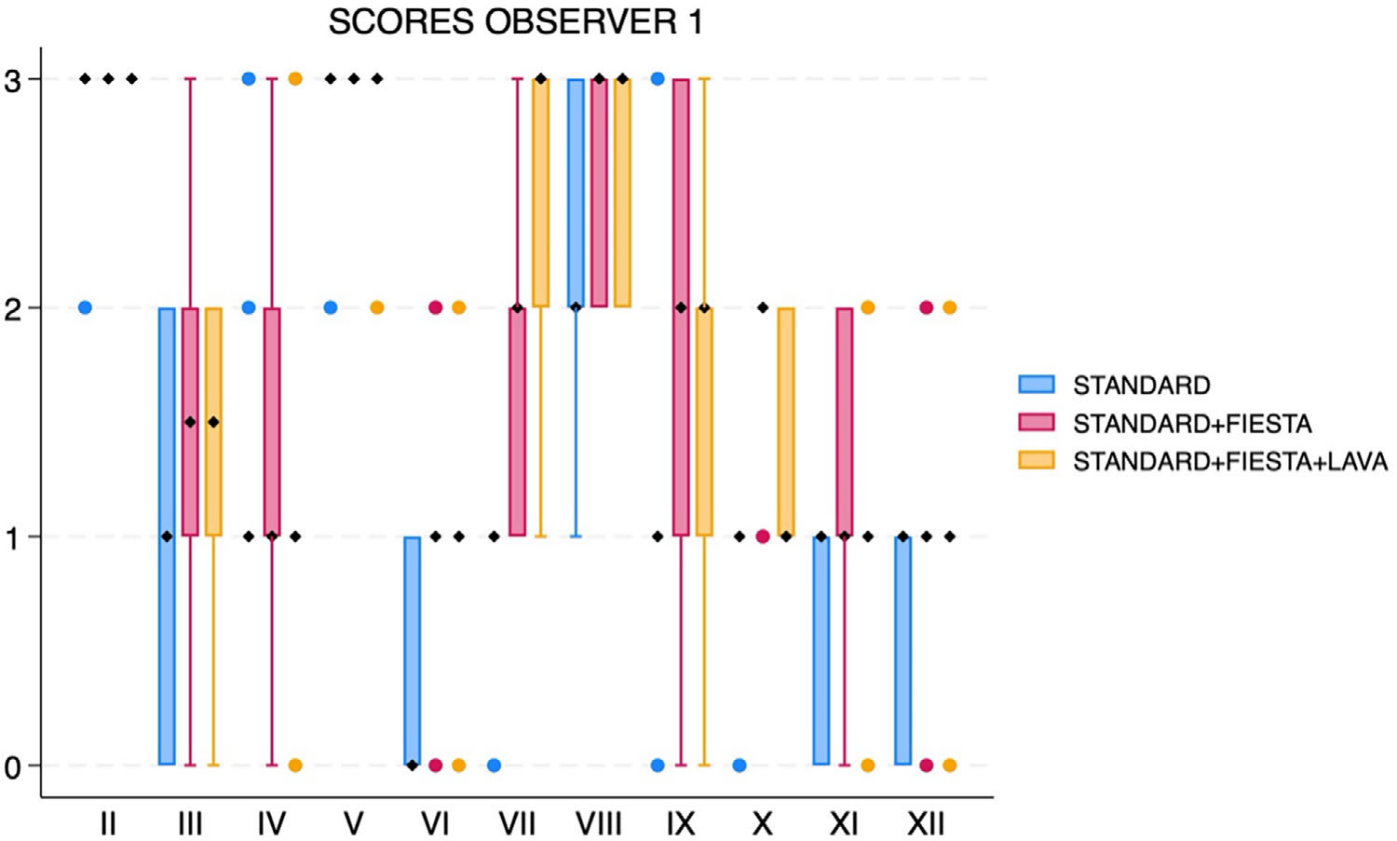
Box and whisker plot showing scores for each cranial nerve, separated by sequences (standard, standard with FIESTA‐C, and standard with FIESTA‐C and LAVA), for observer 1. The black diamonds represent the median values. The boxes represent the interquartile range (25th–75th percentiles), and the whiskers represent the minimum and maximum values. FIESTA, Fast Imaging Employing Steady‐State Acquisition; LAVA, Liver Acquisition with Volume Acceleration.

**FIGURE 2:**
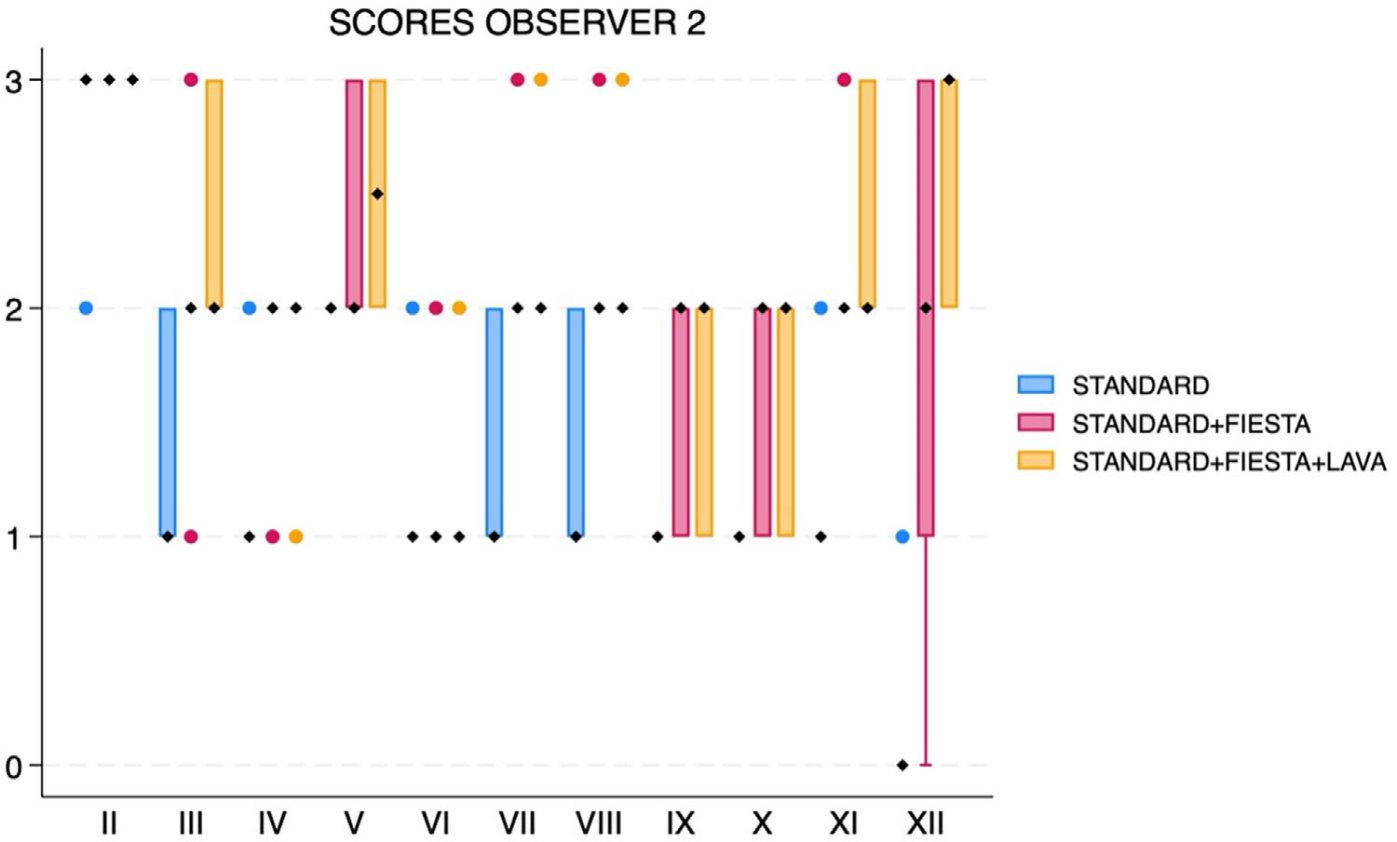
Box and whisker plot showing scores for each cranial nerve, separated by sequences (standard, standard with FIESTA‐C, and standard with FIESTA‐C and LAVA), for observer 2. The black diamonds represent the median values. The boxes represent the interquartile range (25th–75th percentiles), and the whiskers represent the minimum and maximum values. FIESTA, Fast Imaging Employing Steady‐State Acquisition; LAVA, Liver Acquisition with Volume Acceleration.

**FIGURE 3:**
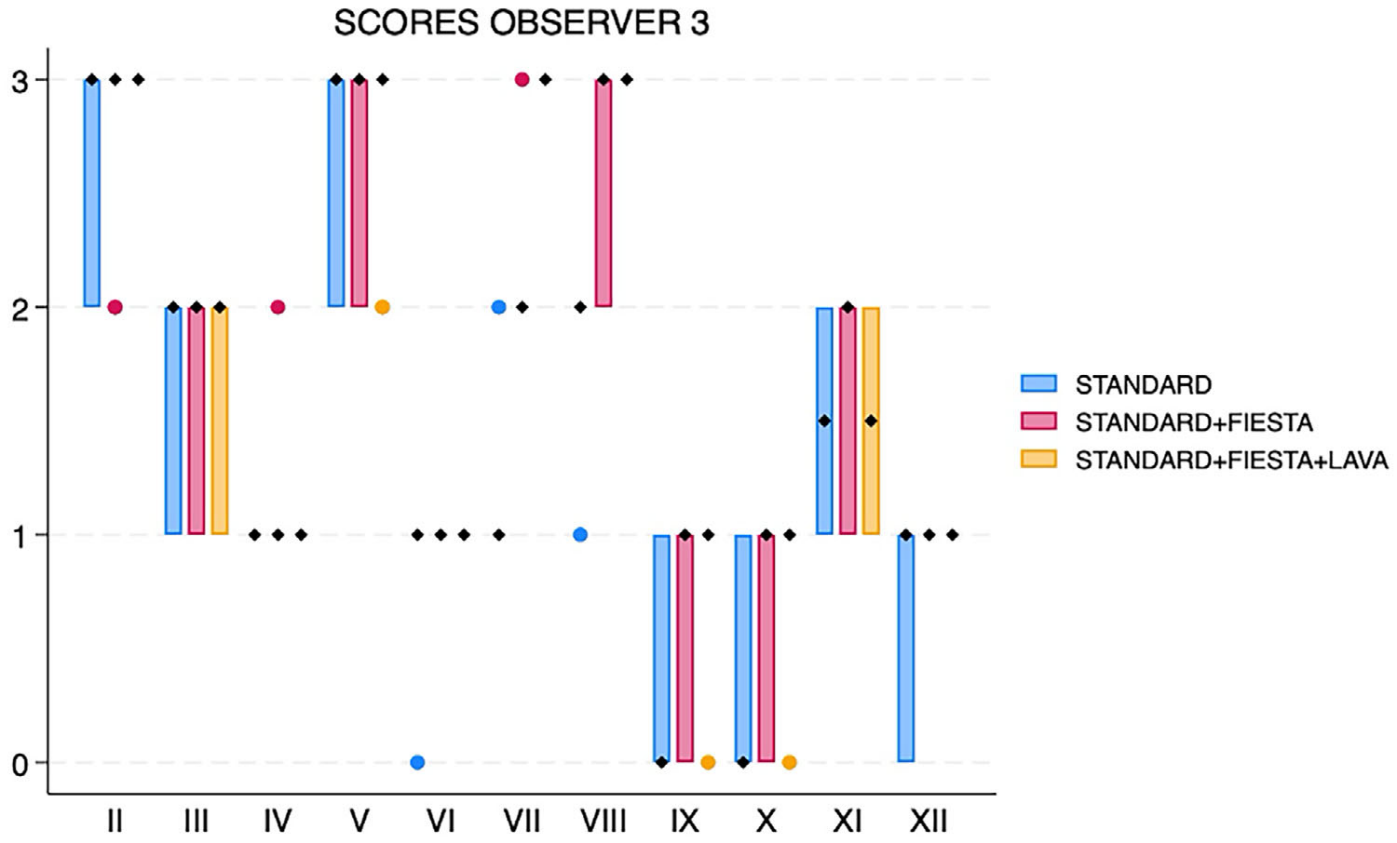
Box and whisker plot showing scores for each cranial nerve, separated by sequences (standard, standard with FIESTA‐C, and standard with FIESTA‐C and LAVA), for observer 3. The black diamonds represent the median values. The boxes represent the interquartile range (25th–75th percentiles), and the whiskers represent the minimum and maximum values. FIESTA, Fast Imaging Employing Steady‐State Acquisition; LAVA, Liver Acquisition with Volume Acceleration.

When adding FIESTA‐C (acquisition time about 9–9.5 min), all three individual observers’ median scores increased for nerves VII, VIII, IX, and X (Figures 1, 2, 3). Median score also increased for two observers for nerves III and XI. Median scores increased for only one observer for nerves IV, VI, and XII. Median scores were unchanged for all observers for nerves II and V.

**FIGURE 4:**
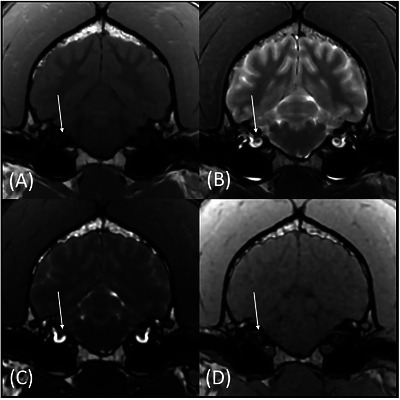
Transverse T1w (A), T2w (B), FIESTA‐C (C), and LAVA (D) magnetic resonance images of the same patient at the level of CN VII (arrows). The CN VII is more conspicuously identified on the FIESTA‐C (C) and LAVA (D) images.

**FIGURE 5:**
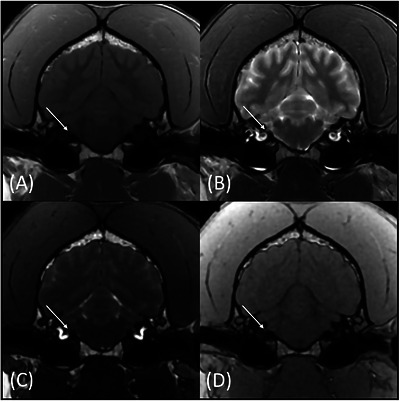
Transverse T1W (A), T2W (B), FIESTA‐C (C), and LAVA (D) magnetic resonance images of the same patient at the level of CN VIII (arrows). The CN VIII is more conspicuously identified on the FIESTA‐C (C) and LAVA (D) images.

**FIGURE 6:**
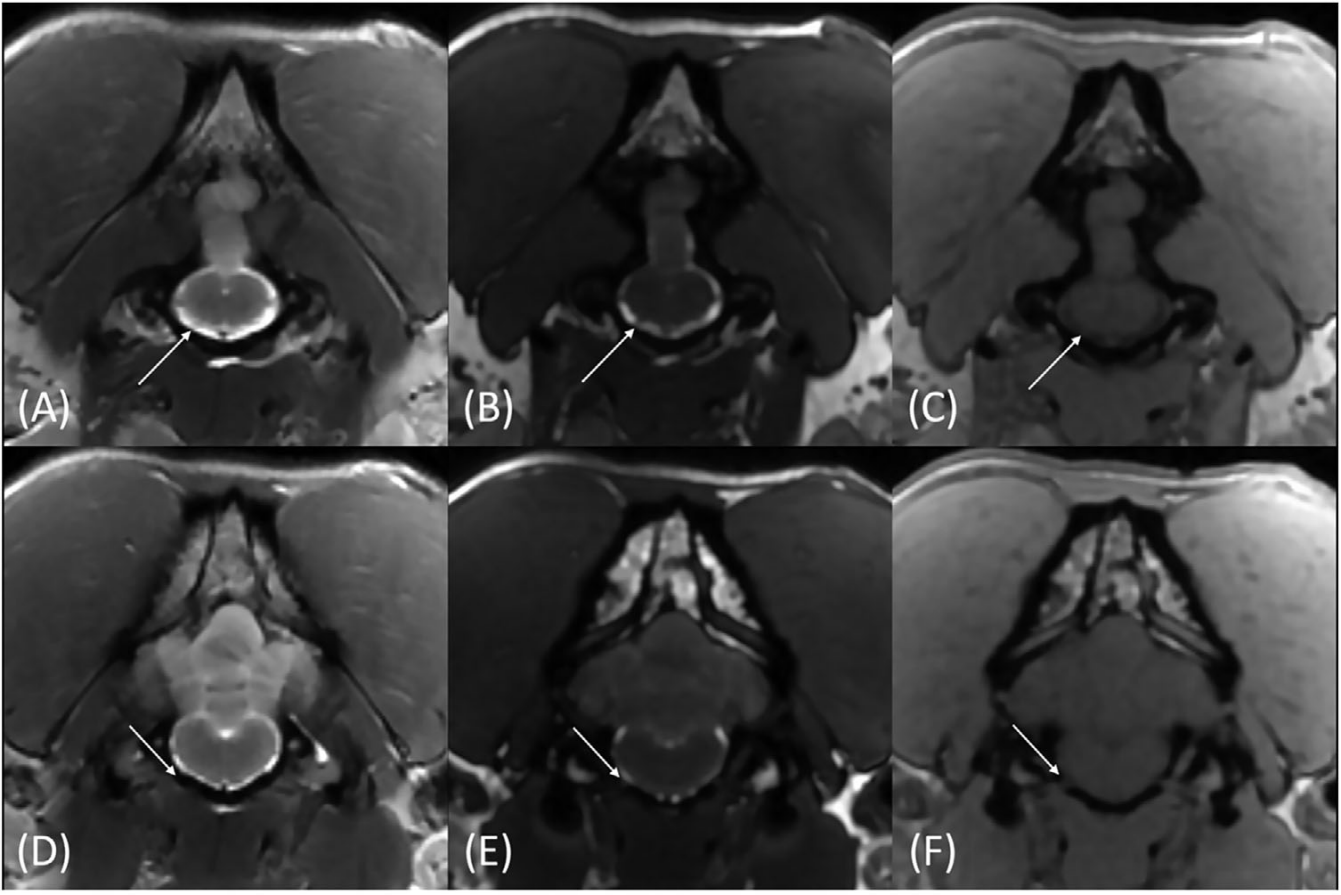
Transverse T2W (A and D), FIESTA‐C (B and E), and LAVA (C and F) magnetic resonance images of the same patient showing the intracranial (A–C) and intraforaminal portions of CN XII through the hypoglossal canal (D–F) (arrows). The very small CN XII is barely visible on the T2W image (A) but more conspicuous on the FIESTA image (B). The hypoglossal canal is better outlined on the LAVA image (arrow, F) and allows for cross‐referencing the nerve on the FIESTA image (arrow, E).

For all three observers, matched‐pairs comparison revealed overall significant score differences for nerves VII (*p* value range 0.0016–0.0063 across all three observers) and VIII (*p* value range 0.0047–0.0265) (Figures 4 and 5). There were significant score differences for only two observers for nerves III (*p* values 0.0082 and 0.047), IX (*p* values 0.0143 and 0.0271), X (*p* values 0.0035 and 0.0143), XI (*p* values 0.0042 and 0.0143), and XII (*p* values 0.0054 and 0.0833) (Figure 6).

The addition of LAVA (acquisition time about 7–8 min) did not significantly improve confidence, except for nerve VII in two observers (*p* = 0.0027 and 0.0466) and nerve XII in one observer (*p* = 0.0271).

**FIGURE 7:**
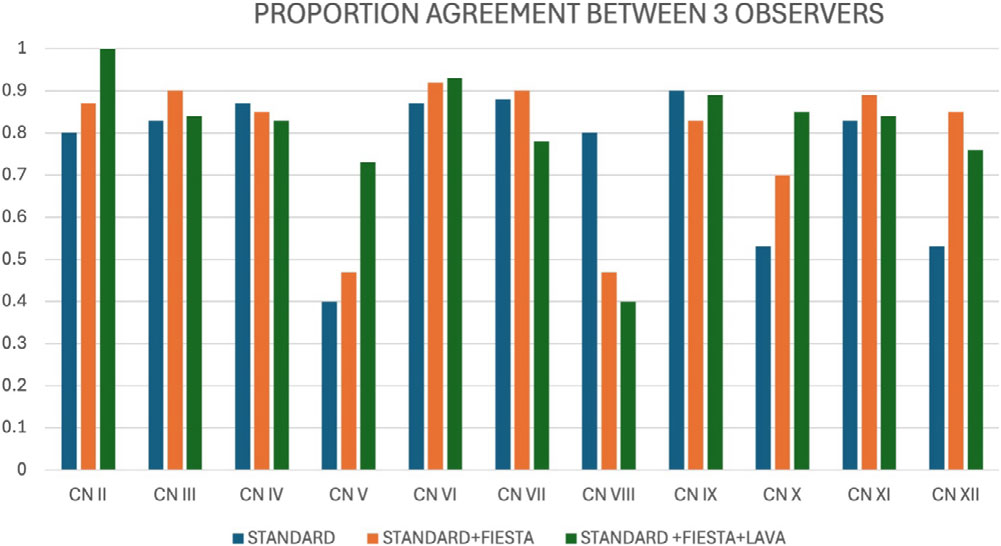
Proportion agreement (0–1 scale) between the three observers for each round of grading. Standard sequences in blue, standard sequences with FIESTA‐C in orange, and standard sequences with FIESTA‐C and LAVA in green.

Overall percent agreement between observers ranged between 40% and 90% (average 74.9%) for standard pulse sequences, 47%–92% (average 78.6%) for standard + FIESTA‐C, and 40%–100% (average 89.5%) for standard + FIESTA‐C + LAVA (Figure 7). On the basis of weighted kappa calculation, interobserver agreement for all three protocols was generally moderate to substantial but only slight to fair for nerves V, VIII, X, and XII with standard pulse sequences; nerves V, VIII, IX, and X for standard + FIESTA‐C; and nerves VII, VIII, and XII for standard + FIESTA‐C + LAVA.

## 4 Discussion

Although the addition of a b‐SSFP sequence did not consistently improve confidence of identification for all nerves, we observed a significant increase in confidence score for some nerves, such as VII and VIII, which are commonly affected by pathology in dogs, when including FIESTA‐C (Figures 4 and 5). Improved visualization of CNs VII and VIII with the addition of FIESTA‐C is notable, as these nerves are commonly described as readily visible on standard MRI sequences. We attributed the improved visualization of these nerves in this study with FIESTA‐C to its higher spatial and contrast resolution and to the scoring scale design, which allowed observers to score nerves higher when more of the nerve's path was visualized, rather than solely in a binary “visible or not” method. Additionally, a significant increase in confidence score was also observed with the addition of b‐SSFP for CN XII, which is often difficult to visualize on standard pulse sequences (Figure 6). Therefore, a b‐SSFP sequence may be a useful addition to a standard MRI protocol when there is clinical concern for CN disease.

The addition of a 3D T1w FSPGR sequence, such as LAVA, may be further beneficial in the identification of CNs VII and XII, though the added value was not observed across all observers. Improvement in scoring may be related to improved visualization of the associated osseous skull foramina (the facial canal and hypoglossal canal, respectively), based on the known imaging characteristics of T1w FSPGR sequences; in turn, this improved foraminal visibility may have enabled better tracing of these nerves from the foramina to their origins. However, the independent contribution of LAVA cannot be determined from the current study design. LAVA can be acquired during the same MRI examination, enabling direct, accurate spatial co‐registration with FIESTA‐C images and facilitating cross‐referencing between sequences. Although CT has been used in some studies as an adjunct to MRI for assessment of CNs [3], short TE 3D T1w FSPGR pulse sequences, similar to LAVA used here, were shown to be a valid alternative to CT for assessment of skull structures in situations where additional CT imaging would be too time‐consuming or cost‐prohibitive [10]. The degree of usefulness of LAVA alone was not evaluated, as LAVA sequences were not scored without concurrent evaluation of FIESTA‐C.

It is noteworthy that confidence levels are highly variable across observers, and interobserver agreement varies widely across nerves and pulse sequence combinations. This is likely multifactorial. Differences in observer experience in MRI interpretation (6, 12, and 21 years) may have contributed, particularly in the interpretation of subtle structures such as small CNs, where confidence thresholds may vary between readers. Additionally, the use of an ordinal scoring system introduces inherent subjectivity, especially when distinguishing between partial and complete visualization. Although visualization and confidence are conceptually distinct, the extent of nerve visualization was used in this study as a practical surrogate for confidence, as more complete and continuous visualization of a nerve generally corresponds to higher interpretive certainty. This approach may oversimplify the relationship between these constructs and could contribute to interobserver variability. The intrinsic difficulty of identifying certain CNs due to their small size, variable course, and limited surrounding CSF likely further contributed to variability. For CN V, variability in scoring may have been further influenced by differences in how observers integrated visualization of its three branches into a single score, as no standardized weighting approach was defined. This may have contributed to lower interobserver agreement for this nerve. The slight decrease in agreement observed for some nerves with the addition of LAVA may be due to increased interpretive complexity when integrating multiple sequences, as well as small shifts in scoring thresholds (e.g., partial vs. complete visualization), which can disproportionately impact agreement metrics in a small sample. Of note is the combination of high percent agreement and lower kappa values observed for some CNs: this may reflect a prevalence effect, where scores cluster toward one end of the ordinal scale. This is a recognized limitation of kappa statistics and may underestimate agreement in such scenarios [13].

Some nerves remain very difficult to clearly identify on MRI, even with the addition of FIESTA‐C and LAVA pulse sequences, such as nerves III, IV, and VI. This is probably due to a combination of their very small diameters as well as the absence of CSF surrounding them in their non‐cisternal portions, such as when they travel through the orbital fissure, rostral to the middle cranial fossa. This is also the case in humans: For example, in an anatomic study, the trochlear nerve (CN IV) was not identified on 3D CISS images, a b‐SSFP pulse sequence analogous to FIESTA‐C, in more than 50% of patients [5]. In that study [5], most other CNs were adequately visualized with 3D CISS, and rates of successful visualization were higher than with T2w fast spin‐echo sequences; that study used slightly smaller voxels (0.35 × 0.69 mm^2^ pixels with 0.7 mm slice thickness) compared to 0.8 × 0.8 × 0.8 mm^3^ in our study. Other imaging parameters used in our study were similar to those used in that human anatomic study. Future research using even smaller voxels may be considered to evaluate whether this would further improve the visualization of canine CNs. Future studies at 3 T may also be considered, as these scanners offer higher signal‐to‐noise ratio and spatial resolution; however, the higher susceptibility to artifacts from bone and air interfaces near the base of the skull may counterbalance these benefits. Finally, diffusion tensor imaging may be considered in the future as an adjunct imaging technique to improve CNs assessment.

There are several limitations to this study. Unconscious bias cannot be eliminated as a contributing factor to the observed improved visualization of some nerves when adding high‐resolution pulse sequences, as the observers were not blinded to the type of pulse sequences they were evaluating. Complete blinding is inherently challenging in this context, as sequences such as FIESTA‐C and LAVA have distinct and readily recognizable image characteristics. Future studies could aim to reduce bias through alternative study designs, such as randomizing the order of case presentation and sequence combinations, or by having observers evaluate sequences independently rather than cumulatively. Increasing the washout period and sample size may also help mitigate recall and learning effects. Although these approaches may not fully eliminate bias, they could reduce its impact and further strengthen the evaluation of these imaging techniques.

The relatively small sample size and the number of statistical comparisons performed may have limited the power to detect smaller differences and increased the risk of both Type I and Type II errors. As such, the findings of this study should be interpreted as exploratory and hypothesis‐generating. For the same reasons, no correction for multiple comparisons was applied.

Evaluation was undertaken in cadaveric patients; therefore, intravenous contrast administration was not feasible. In people, post‐contrast 3D b‐SSFP pulse sequences have been shown to improve the visibility of the intracavernous portions of CNs III, IV, and VI and the ophthalmic and maxillary branches of CN V. This is due to the enhancement in the cavernous venous plexus, which plays a similar role to CSF and allows a better delineation of these CNs on post‐contrast b‐SSFP images [14, 15]. Similarly, post‐contrast 3D‐FIESTA pulse sequence was reported to improve visualization of the jugular foramen nerves (IX, X, and XI) [16]. Therefore, pre‐contrast b‐SSFP sequences and post‐contrast imaging should be considered complementary techniques, each optimizing visualization in different segments of the CN pathways. The present study focused on the added value of pre‐contrast sequences within a standard protocol and did not aim to assess the impact of post‐contrast imaging.

Even though we evaluated images obtained in freshly deceased cadavers, it is unlikely that results would be different in live, anesthetized animals: Although steady‐state pulse sequences are typically very sensitive to motion and flow, FIESTA‐C, with its cycled pulses, provides motion compensation, making it suitable for imaging the cisternal portion of the CNs with no motion or flow artifacts. We only evaluated images in the transverse plane, as this is typically the most useful plane to identify CNs in dogs [3]. However, since b‐SSFP and FSPGR pulse sequences are three‐dimensional, further studies that leverage multiplanar reconstructions to generate oblique images tailored to specific CN paths may also be considered. Although head conformation may influence CNs visualization, it was not specifically accounted for in this study; however, the present study included only 10 dogs, with a heterogeneous breed distribution, resulting in an insufficient sample size to allow meaningful subgroup analysis by skull type (brachycephalic, mesaticephalic, or dolichocephalic). Future studies with larger, more balanced populations could help determine whether skull conformation has a measurable impact on CN visualization with these sequences. Lastly, our sample size for this study was small. Further studies using larger sample sizes may be useful.

Further studies examining patients with clinical CN disease may help identify the utility of b‐SSFP and FSPGR sequences in specific, commonly encountered disease states affecting CNs, such as facial/vestibulocochlear neuritis and trigeminal neuropathies. These sequences are commonly used in human medicine to identify CN pathology and to evaluate their detailed anatomy in disease states, such as vestibulocochlear disease, facial nerve evaluation, and tumor evaluation, including the extent of neoplastic infiltration [8].

In conclusion, adding a b‐SSFP pulse sequence to a standard MRI protocol may help identify certain CNs in dogs, particularly CN VII and CN VIII. The addition of a T1w FSPGR pulse sequence (e.g., 3D LAVA) may provide an inconsistent benefit for some radiologists; however, the benefit must be weighed against the added acquisition and anesthesia time (about 9–9.5 min for 3D FIESTA and 7–8 min for 3D LAVA). Further studies are necessary to determine whether adding these sequences improves the identification of CNs in cases of specific CN pathology and whether post‐contrast imaging would further enhance CN visibility.

## Author Contributions

Conception and design: Rachel Durrwachter and Wilfried Mai. Acquisition of data: Rachel Durrwachter and Wilfried Mai. Analysis and interpretation of data: Rachel Durrwachter, Wilfried Mai, Jennifer Reetz, Alessia Cordella, and Matthew Paek. Drafting the article: Rachel Durrwachter, Wilfried Mai, Jennifer Reetz, Alessia Cordella, and Matthew Paek. Reviewing article for intellectual content: Rachel Durrwachter, Wilfried Mai, Jennifer Reetz, Alessia Cordella, and Matthew Paek. Final approval of the completed article: Rachel Durrwachter, Wilfried Mai, Jennifer Reetz, Alessia Cordella, and Matthew Paek.

## Disclosure

The abstract for this study was presented in poster format at the 2024 ACVR Annual Scientific Meeting in Norfolk, Virginia.

## Ethics Statement

The canine cadavers used in this study died of natural causes or were humanely euthanized for medical reasons; they were donated by the owners to an institutional educational program. Therefore, Institutional Animal Care and Use Committee approval was not necessary.

## Conflicts of Interest

The authors declare no conflicts of interest.

## Data Availability

The data that support the findings of this study are available from the corresponding author upon reasonable request.

## References

[1] A. T. Parry and H. A. Volk , “Imaging the Cranial Nerves,” Veterinary Radiology & Ultrasound: The Official Journal of the American College of Veterinary Radiology and the International Veterinary Radiology Association 52, no. S1 (2011): S32–S41. 21392154 10.1111/j.1740-8261.2010.01783.x

[2] E. Gomes , C. Degueurce , Y. Ruel , R. Dennis , and D. Begon , “Anatomic Study of Cranial Nerve Emergence and Associated Skull Foramina in Cats Using CT and MRI,” Veterinary Radiology & Ultrasound: The Official Journal of the American College of Veterinary Radiology and the International Veterinary Radiology Association 50, no. 4 (2009): 398–403. 19697605 10.1111/j.1740-8261.2009.01556.x

[3] L. Couturier , C. Degueurce , Y. Ruel , et al., “Anatomical Study of Cranial Nerve Emergence and Skull Foramina in the Dog Using Magnetic Resonance Imaging and Computed Tomography,” Veterinary Radiology & Ultrasound: The Official Journal of the American College of Veterinary Radiology and the International Veterinary Radiology Association 46, no. 5 (2005): 375–383. 16250393 10.1111/j.1740-8261.2005.00068.x

[4] R. Gonçalves , F. Malalana , J. F. McConnell , and T. Maddox , “Anatomical Study of Cranial Nerve Emergence and Skull Foramina in the Horse Using Magnetic Resonance Imaging and Computed Tomography,” Veterinary Radiology & Ultrasound: The Official Journal of the American College of Veterinary Radiology and the International Veterinary Radiology Association 56, no. 4 (2015): 391–397. 25832323 10.1111/vru.12256

[5] I. Yousry , S. Camelio , U. Schmid , et al., “Visualization of Cranial Nerves I–XII: Value of 3D CISS and T2‐Weighted FSE Sequences,” European Radiology 10 (2000): 1061–1067. 11003398 10.1007/s003300000452

[6] W. Mai , “Cranial Nerve Diseases,” in Diagnostic MRI in Dogs and Cats, ed. W. Mai (CRC Press, 2018): 326–342.

[7] G. B. Chavhan , P. S. Babyn , B. G. Jankharia , et al., “Steady‐State MR Imaging Sequences: Physics, Classification, and Clinical Applications,” Radiographics 28, no. 4 (2008): 1147–1160. 18635634 10.1148/rg.284075031

[8] J. H. Oh , J. H. Chung , H. J. Min , S. H. Cho , C. W. Park , and S. H. Lee , “Clinical Application of 3D‐FIESTA Image in Patients With Unilateral Inner Ear Symptom,” Korean Journal of Audiology 17, no. 3 (December 2013): 111–117. 24653918 10.7874/kja.2013.17.3.111 PMC3936557

[9] S. Sheth , B. BFt , and E. J. Escott , “Appearance of Normal Cranial Nerves on Steady‐State Free Precession MR Images,” Radiographics 29, no. 4 (2009): 1045–1055. 19605655 10.1148/rg.294085743

[10] M. C. Cicci , E. K. Keenihan , K. Bailey , L. Graham , S. Sommer , and E. B. Cohen , “Short/Ultra‐Short TE MRI Sequences Comparable to CT and Superior to Standard MRI Sequences for Canine Skull Imaging,” Veterinary Radiology & Ultrasound: The Official Journal of the American College of Veterinary Radiology and the International Veterinary Radiology Association 66, no. 3 (May 2025): e70035. 40317775 10.1111/vru.70035 PMC12048040

[11] A. Tauro , J. Jovanovik , C. J. Driver , and C. Rusbridge , “Clinical Application of 3D‐CISS MRI Sequences for Diagnosis and Surgical Planning of Spinal Arachnoid Diverticula and Adhesions in Dogs,” Veterinary and Comparative Orthopaedics and Traumatology 31, no. 2 (February 2018): 83–94. 29534275 10.3415/VCOT-16-12-0169

[12] J. R. Landis and G. G. Koch , “The Measurement of Observer Agreement for Categorical Data,” Biometrics 33, no. 1 (March 1977): 159–174. 843571

[13] T. Byrt , J. Bishop , and J. B. Carlin , “Bias, Prevalence and Kappa,” Journal of Clinical Epidemiology 46, no. 5 (1993): 423–429. 8501467 10.1016/0895-4356(93)90018-v

[14] A. Yagi , N. Sato , A. Taketomi , et al., “Normal Cranial Nerves in the Cavernous Sinuses: Contrast‐Enhanced Three‐Dimensional Constructive Interference in the Steady State MR Imaging,” American Journal of Neuroradiology 26, no. 4 (2005): 946–950. 15814950 PMC7977126

[15] A. M. Blitz , L. L. Macedo , Z. D. Chonka , et al., “High‐Resolution CISS MR Imaging With and Without Contrast for Evaluation of the Upper Cranial Nerves,” Neuroimaging Clinics of North America 24, no. 1 (2014): 17–34. 24210310 10.1016/j.nic.2013.03.021

[16] I. Davagnanam and S. V. Chavda , “Identification of the Normal Jugular Foramen and Lower Cranial Nerve Anatomy: Contrast‐Enhanced 3D Fast Imaging Employing Steady‐State Acquisition MR Imaging,” American Journal of Neuroradiology 29, no. 3 (March 2008): 574–576. 18065504 10.3174/ajnr.A0860 PMC8118882

